# Conserved visual sensitivities across divergent lizard lineages that differ in an ultraviolet sexual signal

**DOI:** 10.1002/ece3.5686

**Published:** 2019-09-27

**Authors:** Caroline M. Dong, Claire A. McLean, Adnan Moussalli, Devi Stuart‐Fox

**Affiliations:** ^1^ School of BioSciences The University of Melbourne Parkville Victoria Australia; ^2^ Sciences Department Museums Victoria Carlton Victoria Australia

**Keywords:** cone opsin, *Ctenophorus decresii*, ddPCR, gene expression, sensory drive, visual ecology

## Abstract

The sensory drive hypothesis predicts the correlated evolution of signaling traits and sensory perception in differing environments. For visual signals, adaptive divergence in both color signals and visual sensitivities between populations may contribute to reproductive isolation and promote speciation, but this has rarely been tested or shown in terrestrial species. We tested whether opsin protein expression differs between divergent lineages of the tawny dragon (*Ctenophorus decresii*) that differ in the presence/absence of an ultraviolet sexual signal. We measured the expression of four retinal cone opsin genes (SWS1, SWS2, RH2, and LWS) using droplet digital PCR. We show that gene expression between lineages does not differ significantly, including the UV wavelength sensitive SWS1. We discuss these results in the context of mounting evidence that visual sensitivities are highly conserved in terrestrial systems. Multiple competing requirements may constrain divergence of visual sensitivities in response to sexual signals. Instead, signal contrast could be increased via alternative mechanisms, such as background selection. Our results contribute to a growing understanding of the roles of visual ecology, phylogeny, and behavior on visual system evolution in reptiles.

## Graphical abstract

The sensory drive hypothesis predicts that the coevolution of signaling traits and sensory perception may contribute to reproductive isolation and speciation. We tested whether opsin protein expression differs between divergent lineages of the tawny dragon (*Ctenophorus decresii*) that differ in the presence/absence of an ultraviolet sexual signal. We found that gene expression does not differ and discuss these results in the context of mounting evidence that visual sensitivities are highly conserved in terrestrial systems. Photo credit: Adam Elliott.

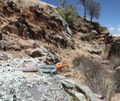

## 1 INTRODUCTION

When a species exists across habitats of varying physical and structural properties, communication systems may adapt to the local environment to optimize the efficacy of signal transmission and interpretation (Endler, 1992, 1993a). This idea is central to the sensory drive hypothesis, which proposes a correlated divergence in signaling traits (e.g., color), sensory perception (e.g., vision), and mate preferences (Endler & Basolo, 1998). The scale and consistency of adaptive divergence in mating signals and perception can influence whether it leads to speciation or the maintenance of color variation within the species (Chunco, McKinnon, & Servedio, 2007; Gray & McKinnon, 2007; Seehausen et al., 2008). Despite more than 25 years of research on sensory drive, the majority of work has focused on aquatic systems, where ambient illumination varies substantially and consistently (reviewed in Cummings & Endler, 2018). Furthermore, most evidence for tuning of color vision to different color signals comes from taxonomic groups that show substantial variation in the number and spectral sensitivities of photoreceptors, such as butterflies and teleost fishes (Bernard & Remington, 1991; Briscoe et al., 2010; Carleton, Parry, Bowmaker, Hunt, & Seehausen, 2005; Hoffmann et al., 2007; Miyagi et al., 2012; Sison‐Mangus, 2006).

In many groups, including birds and reptiles, the number and spectral sensitivities of photoreceptors are highly conserved. However, visual sensitivities can be influenced by the relative proportion of different photoreceptor types (and therefore cone opsin expression) in the retina (reviewed in Carleton, 2014). For example, in New World warblers (Parulidae), relative opsin expression is associated with plumage dichromatism and light environment (Bloch, 2015). Similarly, a high abundance of ultraviolet (UV) sensitive cones have been associated with the presence of a UV signal in a lizard (Fleishman, Loew, & Whiting, 2011). Opsin gene expression may evolve more readily in response to varying selection than opsin spectral tuning (i.e., changing the wavelength of peak photoreceptor sensitivity, *λ*_max_). However, evidence for an association between visual signals and color vision achieved by modifying the relative gene expression of cone opsins is currently limited in terrestrial species (Bloch, Morrow, Chang, & Price, 2015; Coyle, Hart, Carleton, & Borgia, 2012; Tseng et al., 2018; Yewers et al., 2015).

**Figure 1:**
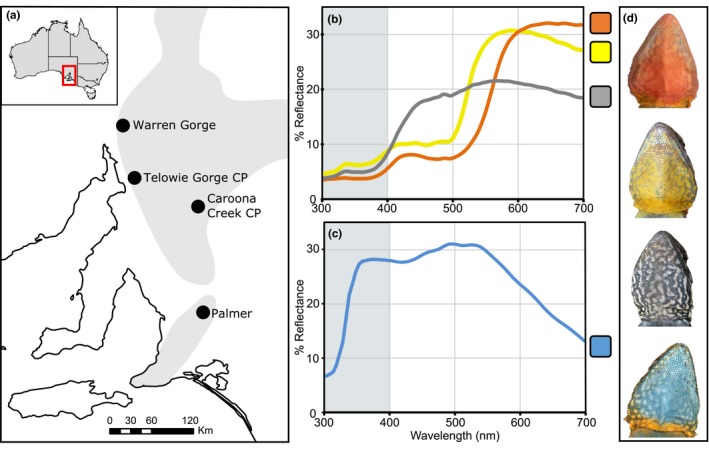
Sampling localities and male throat colors of the tawny dragon (*Ctenophorus decresii*). (a) Map showing localities in the northern and southern lineages. Elevated rocky ranges are shaded in gray. Average reflectance of male throat colors found in the (b) northern lineage: orange, yellow, gray, and in the (c) southern lineage: blue. Ultraviolet wavelengths are highlighted in gray; (d) Representative male throats for each color

The tawny dragon lizard (*Ctenophorus decresii*; Duméril & Bibron, 1837) is a good candidate for examining changes in visual sensitivity associated with divergence of a sexual signal and the signaling environment. The species comprises two genetically and phenotypically distinct lineages which differ markedly in a sexual color signal, male throat coloration (Figure 1). Northern lineage males are polymorphic with four discrete throat morphs: orange, yellow, yellow with an orange center, and gray (Teasdale, Stevens, & Stuart‐Fox, 2013), all of which lack significant UV reflectance. By contrast, southern lineage males are monomorphic with UV‐blue throats with a consistent UV reflectance peak (McLean, Stuart‐Fox, & Moussalli, 2014). This throat coloration is prominently displayed during territorial and courtship displays involving head‐bobbing and push‐ups (Gibbons, 1979, 1977; Osborne, Umbers, Backwell, & Keogh, 2012; Stuart‐Fox & Johnston, 2005) and is locally adapted to increase conspicuousness against the predominant background colors of native lichen in their respective ranges (McLean, Moussalli, & Stuart‐Fox, 2014). The northern lineage is primarily found in semi‐arid sparsely vegetated habitats, whereas the southern lineage occurs in wetter, temperate, more vegetated habitats (Houston, 1974).

Characteristic of diurnal lizards, *C. decresii* has tetrachromatic vision with UV sensitive (SWS1; 364–383 nm), short‐wavelength sensitive (SWS2; 440–467 nm), medium‐wavelength sensitive (rod‐like cone opsin RH2; 483–501 nm), and long‐wavelength sensitive (LWS; 560–625 nm) cone opsins and one rod opsin (RH1; Yewers et al., 2015). There are no significant differences in the absorption spectra of visual pigments between lineages of *C. decresii*, nor in amino acid sequences of opsin genes (Yewers et al., 2015). However, color discrimination may be fine‐tuned by differences in the relative proportion of photoreceptor types rather than shifts in their peak wavelength sensitivities. Given the occurrence of locally adapted throat coloration in *C. decresii*, we hypothesized that the lineages may differ in visual sensitivities via the relative expression of cone opsin genes. Specifically, we predicted that the southern lineage would exhibit higher expression of the UV sensitive SWS1 opsin gene due to the UV reflectance peak found on male throats.

## 2 METHODS

### 2.1 Animals

We analyzed the visual sensitivities of seven northern lineage (six males and one female) and nine southern lineage (seven males and two females) individuals (Table 1). We focused primarily on males because in *C. decresii*, males compete for access to females and opportunities for female male choice appear to be limited, as is generally the case in lizards (Lailvaux & Irschick, 2006; Lebas & Marshall, 2001; Olsson & Madsen, 1995; Smith & Zucker, 1997; Tokarz, 1995). Male–male interactions are therefore the strongest determinants of mating success in territorial lizards (Gullberg, Olsson, & Tegelstrom, 1997; Simon, 2011; Stamps & Krishnan, 1997; Tokarz, 1998); however, we included a subset of females for comparison. Northern lineage individuals were wild‐caught by noosing or by hand from Caroona Creek Conservation Park (longitude: 139.103, latitude: −33.443), Telowie Gorge Conservation Park (longitude: 138.106, latitude: −33.023), or Warren Gorge, South Australia (longitude: 137.995, latitude: −32.183). Southern lineage individuals were captured from Palmer, South Australia (longitude: 139.159, latitude: −34.851) or were hatched from eggs laid by gravid females captured from Palmer and raised in captivity to sexual maturity (>1 year; Gibbons, 1977). The subjects were housed individually in opaque plastic tubs (55 × 34 × 38 cm [L × W × D]) with sand and two terracotta tiles to provide shelter and a basking platform. Lizards were kept in captivity between 3 months to 1.5 years at The University of Melbourne, Melbourne and Deakin University, Geelong, Australia. They were provided with artificial lighting and seasonal photoperiods to approximate natural conditions, including UV lighting and a heat lamp for thermoregulation. Lizards were fed crickets *ad libitum* and misted with water 3 days a week. Although individuals differed slightly in age and period of time in captivity, all were sexually mature at the time of sampling and suitable to investigate consistent differences in visual sensitivities between lineages. This research was conducted with approval from the Department of Environment, Water and Natural Resources, South Australia (permit nos. E25861‐4 and Q26428‐3), the Department of Environment, Land, Water and Planning, Victoria (permit nos. 10007000 and 10007751) and with approval by the University of Melbourne Animals Ethics Committee (approval nos. 1312927.1 and 1413220.4), the Wildlife Ethics Committee of South Australia (approval nos. 35/2013 and 25/2015), and the Deakin Animal Ethics Committee (project no. G39‐2013).

**Table 1:** Details of all individuals used in the study, including their lineage, sex, capture locality, housing facility, and length of housing (months)

ID	Lineage	Sex	Locality	Housing	Months
NM1	N	M	Telowie Gorge CP	Deakin University	3
NM2	N	M	Telowie Gorge CP	Deakin University	3
NM3	N	M	Caroona Creek CP	Deakin University	3
NM4	N	M	Telowie Gorge CP	Deakin University	3
NM5	N	M	Warren Gorge	The University of Melbourne	3
NM6	N	M	Caroona Creek CP	The University of Melbourne	18
NF1	N	F	Caroona Creek CP	The University of Melbourne	18
SM1	S	M	Palmer	Deakin University	3
SM2	S	M	Palmer	Deakin University	3
SM3	S	M	Palmer	Deakin University	3
SM4	S	M	Captive‐bred (Palmer)	The University of Melbourne	6
SM5	S	M	Captive‐bred (Palmer)	The University of Melbourne	6
SM6	S	M	Captive‐bred (Palmer)	The University of Melbourne	6
SM7	S	M	Captive‐bred (Palmer)	The University of Melbourne	6
SF1	S	F	Palmer	The University of Melbourne	8
SF2	S	F	Palmer	The University of Melbourne	4

### 2.2 Quantification of opsin expression

Following humane euthanasia, eyeballs were immediately removed, hemisected, and stored in RNAlater (Ambion Inc.) at −20°C. Whole retinas were dissected for each individual before being disrupted and homogenized using the TissueLyser II with 3 mm stainless steel beads (Qiagen). Total RNA was extracted using an RNeasy Mini Kit (Qiagen) and quantified on a 220 TapeStation (RIN scores >8.0; Agilent). For each sample, 200 ng of total RNA was reverse‐transcribed to cDNA using a qPCRBIO cDNA Synthesis Kit (PCR Biosystems) and opsin gene expression was measured using droplet digital PCR (ddPCR), a method of digital PCR that implements a water‐in‐oil droplet system using the Bio‐Rad QX100 system (detailed methods in Hindson et al., 2011). This method provides absolute quantification of copies of the PCR target, without the use of a standard curve, and has been shown to have comparable sensitivity to real‐time PCR with the advantages of greater precision and improved reproducibility (Hindson et al., 2013). We used a 25 ul reaction mix containing cDNA, primers (900 nM each), probe (250 nM), and ddPCR Supermix for Probes (no dUTP, Bio‐Rad). Using the QX100 Droplet Generator, this mixture is partitioned into 20,000 droplets and each droplet becomes an independent amplification event. The amplification protocol was as follows: 10 min at 95°C, 40 cycles of 30 s at 94°C and 1 min at 57°C, followed by 10 min at 98°C, ramp rate set to 2.5°C/s. Following amplification on a standard thermal cycler, droplets are analyzed on the QX100 Droplet Reader which counts the number of droplets containing the PCR target (positive) and droplets without (negative) in each sample. These data were analyzed with QuantaSoft Analysis Pro v1.0.596 (Bio‐Rad) which uses a Poisson distribution to determine the absolute template quantity (copies per µl). We then calculated relative opsin gene expression as a percentage of total cone opsin genes expressed for each individual.

We designed primers and probes for the four cone opsin genes (SWS1, SWS2, RH2, LWS; Table 2) based on published opsin sequences from retinal transcriptomes of *C. decresii* (Yewers et al., 2015) using Primer3 v4.1.0 (Koressaar & Remm, 2007; Untergasser et al., 2012). Additionally, primer binding sites were designed to span an exon–exon junction to avoid amplification of genomic DNA and amplified short fragments (75–200 bp). Intron–exon junctions were identified by aligning opsin gene sequences with the draft genome for *C. decresii* (McLean, Stuart‐Fox, and Moussalli, unpublished data) using Exonerate v2.2.0 (Slater & Birney, 2005). A single mix of primers and probes was used for each opsin gene for the length of the experiment.

**Table 2:** Forward primer, probe, and reverse primer sequences for each of the four cone opsins

Opsin	Forward primer	Reverse primer	Probe
SWS1	ACA GTT CAG GGC TTG CAT TA	TGG AAG AGA CAG AGG AGA CC	/56‐FAM/ACC CAT GAC/ZEN/AGA TGA ATC CGA CGT /3IABkFQ/
SWS2	CAA GGC CTC CTC AGT TTA CAA	GAA CTC GAA ACA TCA TCT TCA TCA C	/56‐FAM/TGA ACA AGC/ZEN/AGT TCC GCT CCT GTA /3IABkFQ/
LWS	GCT GTC ATT ATC CTC TGC TAC C	CAC TTC CCT TTC AGC CTT CT	/56‐FAM/CAG CAA CCG/ZEN/CAC GAA TAG CCA AC/3IABkFQ/
RH2	CTC AAA GAG TTC GTC CCT CTA TAA	GTT CTT GCC ACA GCA GAT TG	/56‐FAM/CGT CCT CAT/ZEN/GAA CAA GCA GTT CCG T/3IABkFQ/

### 2.3 Statistical analyses

We used a linear mixed‐effects model to examine the effects of lineage on opsin gene expression. Specifically, we had lineage, gene, and their interaction as fixed terms and included months in captivity, lizard ID, and age as random‐effect terms (opsin expression~lineage + gene + lineage*gene + (1|months) + (1|ID)) + (1|age). We used our model to examine the normality of residuals and found significant departure (Shapiro–Wilk normality test, *p* < .0001). We log‐transformed the data and confirmed normality (*p* = .30). The random‐effect terms each accounted for a negligible amount of variability in the model (<0.05 total variance). Further, we repeated this analysis on a dataset comprising only males (excluding females; opsin expression~lineage + gene + lineage*gene + (1|months) + (1|ID) + (1|age)). Statistical tests were performed in R v3.3 (R Core Development Team, 2017) with the packages *effects* (Fox, 2003), *lme4* (Bates, Mächler, Bolker, & Walker, 2014), and *lmerTest* (Kuznetsova, 2017). We conducted a post hoc power analysis in the program G*Power v3.1.9.4 (Faul, Erdfelder, Buchner, & Lang, 2009; Faul, Erdfelder, Lang, & Buchner, 2007), and determined that the datasets comprising all samples and adult males had powers of 0.94 and 0.86, respectively. A power of 0.80 is generally regarded as an appropriate level of statistical power (Cohen, 1988).

## 3 RESULTS

**Figure 2:**
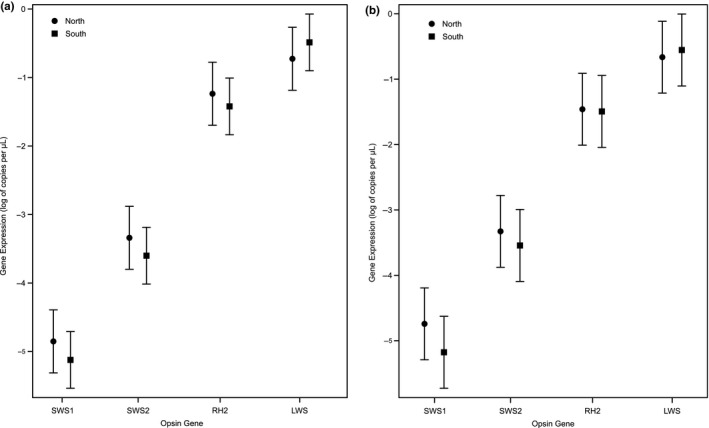
Mean relative expressions (copies per µl) of the four cone opsin genes in the northern and southern lineages for (a) all individuals and (b) a subset of only males. 95% confidence bounds were calculated using parameters estimated from the fitted model

There were no significant differences in gene expression of individual cone opsins between the northern and southern lineages (*p* > .05; Table 3). For the full dataset, the mean relative gene expression ± *SE* for the northern lineage was 0.011 ± 0.007, 0.044 ± 0.022, 0.373 ± 0.224, 0.572 ± 0.201 (SWS1, SWS2, RH2, LWS, respectively). Likewise for the southern lineage, mean relative gene expression ± *SE* was 0.007 ± 0.002, 0.032 ± 0.013, 0.286 ± 0.129, 0.675 ± 0.124 (Figure 2a). We found significant differences between opsin genes (*p* < .0001; Table 3). The relative expression patterns for the four opsin genes were similar between lineages with SWS1 as the lowest expressed gene, followed by SWS2, RH2, and LWS as the most highly expressed gene (northern = 1:4:33.9:52, southern = 1:4.6:40.1:96.4). Analysis of the subset of males recovered qualitatively similar results with no significant differences in expression of each cone opsin between the northern and southern lineages (*p* > .05; Table 3). The mean relative expression ± *SE* for the northern lineage was 0.013 ± 0.006, 0.047 ± 0.021, 0.310 ± 0.164, 0.630 ± 0.142; expression for the southern lineage was 0.007 ± 0.002, 0.036 ± 0.014, 0.286 ± 0.145, 0.671 ± 0.139 (SWS1, SWS2, RH2, LWS, respectively; Figure 2b). Relative expression patterns were also similar between lineages and to the full dataset (northern = 1:3.7:24: 48.7, southern = 1:5.2:41.6:97.4).

**Table 3:** Results of linear mixed‐effects models on all individuals (*N* = 16) and a subset of only males (*N* = 13), statistically significant values are italicized

	All Individuals			Males		
	*df*	*F*	*p*	*df*	*F*	*p*
Lineage	1	0.59	.448	1	0.78	.382
Gene	3	275.72	*<.0001*	3	206.94	*<.0001*
Lineage × Gene	3	1.0	.397	3	0.75	.530

## 4 DISCUSSION

The coevolution of color signals and vision is central to the sensory drive hypothesis, a key proposed mechanism of adaptive divergence and speciation. We examined visual sensitivities of *C. decresii* and found no evidence that relative opsin gene expression levels correspond to divergence between lineages which differ in the presence/absence of a UV sexual signal. We found differences in expression between the four opsin genes; this was similar to the relative expression estimated from transcriptome sequencing in *C. decresii* wherein SWS1 and LWS were the lowest and highest expressed genes but small sample sizes limited further statistical inferences (northern = 1:2.4:2.5:31, *N* = 1; southern = 1.1:2.1:1: 19, *N* = 1; SWS1:SWS2:RH2:LWS; Yewers et al., 2015). A similar hierarchy has been found in the abundance of visual pigments in other squamate taxa (Barbour et al., 2002; Bowmaker, Loew, & Ott, 2005; Tseng et al., 2018). Our results support the hypothesis that diurnal lizards share a highly conserved ancestral pattern of tetrachromatic vision extending into the ultraviolet spectrum as characterized in the families Agamidae (Barbour et al., 2002; Yewers et al., 2015), Chamaeleonidae (Bowmaker et al., 2005), Cordylidae (Fleishman et al., 2011), Dactyloidae (Kawamura & Yokoyama, 1998; Loew, Fleishman, Foster, & Provencio, 2002; Provencio, Loew, & Foster, 1992), and Lacertidae (de Lanuza & Font, 2014). In general, reptiles have retained the set of opsin genes (SWS1, SWS2, RH2, LWS, RH1) inferred to have been present in the ancestral vertebrate (Cronin, Johnsen, Marshall, & Warrant, 2014).

These findings add to the growing body of evidence suggesting that the coevolution of color signals and visual systems is rare in terrestrial systems (Lind, Henze, Kelber, & Osorio, 2017). The strength of selection on visual sensitivities to optimize signal perception is likely to be weaker in terrestrial than aquatic systems because terrestrial light environments are less distinct and more variable over time and space whereas aquatic systems vary more steeply and consistently in background radiance (Chiao, Vorobyev, Cronin, & Osorio, 2000; Endler, 1993b; Goldsmith, 1990; Levine & MacNichol, 1979). Although the lineages of *C. decresii* differ in broad aspects of their habitat (aridity and vegetation cover), both prefer brightly lit open perches and irradiance spectra from full sun conditions are similar across northern and southern localities (McLean, Moussalli, et al., 2014). Moreover, the magnitude of changes in irradiance over a diurnal cycle are likely to be much greater than the overall variation in light conditions between lineages (Endler, 1993b).

The greater variability of terrestrial environments allows for behavioral adjustments to select backgrounds with spectral properties which optimize signal contrast. In *C. decresii*, adults are almost exclusively found on rocks and the colors of the native rock and lichen differs between lineages. Gray and pink rocks with orange lichen are found in the range of the southern lineage whereas orange rocks with pale green lichen are principally found in the range of the northern lineage. The throat coloration of each lineage has been shown to be more chromatically conspicuous against the predominant native lichen color background and less conspicuous against native rock color backgrounds (McLean, Moussalli, et al., 2014). This suggests that individuals could select a background substrate to maximize contrast or crypsis. There is evidence from various terrestrial taxa of local adaptation and/or that individuals can select or modify their environment to alter their conspicuousness (Bortolotti, Stoffel, & Galvan, 2011; Endler & Day, 2006; Endler & Thery, 1996; Gunderson, Fleishman, & Leal, 2018; Heindl & Winkler, 2003; Klomp, Stuart‐Fox, Das, & Ord, 2017; Leal & Fleishman, 2002; Macedonia, 2001; Marshall, Philpot, & Stevens, 2016; Nafus et al., 2015; Sicsú, Manica, Maia, & Macedo, 2013; Uy & Endler, 2004). For example, two closely related species of *Anolis* lizards, *A. cooki*, and *A. cristatellus*, differ markedly in dewlap UV reflectance and have adaptively diverged in microhabitat preference to select light conditions that maximize signal contrast (Leal & Fleishman, 2002). However, similar to our findings, there are no significant differences in spectral sensitivity between ecologically diverse species of *Anolis* with varying dewlap colorations (Fleishman et al., 1997; Loew et al., 2002).

Other ecological factors may generate selection pressures on aspects of the visual system that constrain divergence in response to sexual signals. Visual systems have evolved to accommodate a broad gauntlet of activities critical to survival and fitness. Lizards rely primarily on visual signals at longer distances (López & Martín, 2001; López, Martín, & Cuadrado, 2002; Whiting, Webb, & Keogh, 2009), and individuals must detect and interpret many objects in their environment including suitable shelter, potential rivals and mates, predators, and prey. The lineages of *C. decresii* share similar predominantly avian predators and a generalist diet of insects (Gibbons, 1977), which often have color patterns that reflect or absorb selectively in the UV spectrum (Théry & Gomez, 2010). Additionally, males of both lineages may need to interpret UV signals in females. In the closely related species *Ctenophorus ornatus*, males prefer females with higher throat UV reflectance (LeBas & Marshall, 2000). Female *C. decresii* have UV‐white throats and some exhibit yellow coloration on the throat and sides of the abdomen during the breeding season (Dong and Stuart‐Fox, Personal Observation). The function of female coloration in *C. decresii* is yet to be determined, but could indicate receptivity as in other closely related species (Stuart‐Fox & Goode, 2014). Thus, there may be stronger selection for visual sensitivities that optimize performance across a variety of tasks, than for those tuned to a specific sexually selected signal.

Visual sensitivities can vary in relation to a range of intrinsic and extrinsic factors, such as carotenoid availability, sex steroids, and light environment. For example, testosterone may regulate opsin expression in the sexually dimorphic green‐spotted grass lizard *Takydromus viridipunctatus* (Tseng et al., 2018). However, the great majority of evidence for environmental regulation of opsin expression comes from fish, which experience drastic changes in ambient light environment with habitat changes during ontogeny (Bowmaker & Kunz, 1987; Cheng, 2004; Cottrill et al., 2009; Shand, Archer, & Collin, 1999; Shand et al., 2008; Shand, Hart, Thomas, & Partridge, 2002) and even across diurnal cycles (Johnson, Stanis, & Fuller, 2013). It is possible that environmental factors contributed variation to our data; however, there were no differences in opsin gene expression between sexes and these groups do not differ in diet or habitat preferences (Gibbons, 1977).

## 5 CONCLUSIONS

In summary, we found no evidence for divergence in visual sensitivities between lineages of *C. decresii* that differ in the presence of a UV sexual signal. Our findings are consistent with weaker divergent selection on visual sensitivities within and between closely related species in terrestrial systems. The lack of divergence in visual sensitivities between lineages of *C. decresii* can likely be attributed to similar selection on color vision imposed by the abiotic and biotic environment. Instead, male *C. decresii* may increase conspicuousness of their throat coloration to conspecifics by selecting contrasting backgrounds. By testing for opsin expression divergence in a terrestrial reptile, our study contributes to a growing understanding of broad‐scale patterns in the coevolution of signals and sensory systems, and how they differ between taxonomic groups and environments.

## CONFLICT OF INTEREST

The authors declare that they have no competing interests.

## AUTHOR CONTRIBUTIONS

All authors contributed to study design and statistical analysis. CAM contributed to fieldwork. CMD designed primers, conducted ddPCR data collection, and wrote the manuscript. All authors edited and approved the final manuscript.

## Data Availability

The dataset and R code are available from Dryad: https://doi.org/10.5061/dryad.0fg8g9h
